# Evolution Acts on Enhancer Organization to Fine-Tune Gradient Threshold Readouts

**DOI:** 10.1371/journal.pbio.0060263

**Published:** 2008-11-04

**Authors:** Justin Crocker, Yoichiro Tamori, Albert Erives

**Affiliations:** Department of Biological Sciences, Dartmouth College, Hanover, New Hampshire, United States of America; University of California, Berkeley, United States of America

## Abstract

The elucidation of principles governing evolution of gene regulatory sequence is critical to the study of metazoan diversification. We are therefore exploring the structure and organizational constraints of regulatory sequences by studying functionally equivalent *cis*-regulatory modules (CRMs) that have been evolving in parallel across several loci. Such an independent dataset allows a multi-locus study that is not hampered by nonfunctional or constrained homology. The neurogenic ectoderm enhancers (NEEs) of Drosophila melanogaster are one such class of coordinately regulated CRMs. The NEEs share a common organization of binding sites and as a set would be useful to study the relationship between CRM organization and CRM activity across evolving lineages. We used the D. melanogaster transgenic system to screen for functional adaptations in the NEEs from divergent drosophilid species. We show that the individual NEE modules across a genome in any one lineage have independently evolved adaptations to compensate for lineage-specific developmental and/or genomic changes. Specifically, we show that both the site composition and the site organization of NEEs have been finely tuned by distinct, lineage-specific selection pressures in each of the three divergent species that we have examined: D. melanogaster, D. pseudoobscura, and D. virilis. Furthermore, by precisely altering the organization of NEEs with different morphogen gradient threshold readouts, we show that CRM organizational evolution is sufficient for explaining changes in enhancer activity. Thus, evolution can act on CRM organization to fine-tune morphogen gradient threshold readouts over a wide dynamic range. Our study demonstrates that equivalence classes of CRMs are powerful tools for detecting lineage-specific adaptations by gene regulatory sequences.

## Author Summary

The regulatory control of genes allows an organism to generate a diversity of cell types throughout its body. Gene regulation involves specialized DNA sequences called transcriptional enhancers that increase the expression of genes in specific places and times. Enhancers contain clusters of specific DNA sequences that are uniquely recognized by DNA binding proteins, whose activities are also regulated in space and time. The critical role that DNA enhancers play in generating the diversity of cell types within a single organism suggests that changes in these DNA sequences may also underlie the diversity of organismal forms produced by evolution. However, few examples linking specific changes in enhancer sequences to functional adaptations have been documented. We studied a group of neuro-embryonic enhancers that turn on a certain group of genes in different fruit fly species that have been diverging from each other for ∼50 million years. Each species has experienced unique changes in its protein-coding sequences, gene regulatory sequences, egg morphology, and developmental timing. We found that the organizational spacing between the protein binding sites in these enhancers has evolved in a manner that is consistent with functional adaptations compensating for the dynamic and idiosyncratic evolutionary history of each species.

## Abstract 3

Characterizing neuroectodermal enhancers located throughout the genomes of three*Drosophila* species reveals that each enhancer evolved functional adaptations that compensate for the evolutionary changes specific to each lineage.

## Introduction

The state of a biological cell can be defined by the combined transcriptional status of each gene in a genome. Developmental systems specify cell state by regulating transitions between states. The regulatory logic for these state transitions is encoded in *cis*-regulatory DNA sequences, which specify the transcriptional activity of each gene [1,2]. Each gene may be controlled by multiple locus-specific, independently acting *cis*-regulatory modules (CRMs), which function as transcriptional enhancers, silencers, and insulators [3,4]. Such a set of CRMs can function collectively to sculpt a robust, complex spatiotemporal expression pattern [5]. Because of the critical role that CRMs play in specifying the transcriptional states of a cell, they have been proposed to be a primary target of natural selection [6–20]. Nonetheless, the relative importance of *cis*-regulatory versus protein-coding evolution has been debated because of a relative deficit of specific examples of functional CRM evolution [21].

Some important functional and evolutionary properties of CRMs have been elucidated. For example, enhancers possess switch-like properties that respond to well-defined physiological conditions [22–25], and generally enhancers can drive expression of a heterologous locus when placed almost anywhere into that locus [2]. Each CRM itself is a DNA segment of around 200 to 400 bp long that is composed of clustered binding sites for cooperative and competitive *trans*-acting factors that interact with the DNA. The elements constituting a CRM can arise rapidly anywhere in a gene locus in response to selection [9,17]. Furthermore, slightly deleterious mutations of binding sites in a CRM can be stabilized by the selection of compensatory sites elsewhere in the same CRM [26]. All of these properties of CRMs clearly establish that the evolutionary histories of such DNA sequences are unlike the evolution of protein-coding sequences. However, little is currently known about how evolutionary forces operate on the internal structure of CRMs simply because the organizational constraints of such sequences have not been fully explored.

To address the role of organizational constraints in CRM evolution, we have used the sequenced genomes for three different *Drosophila* species [27], which have been diverging for ∼50 million years [28,29]. These lineages have experienced divergent evolutionary pressures related to lineage-specific ecological life histories. Specific morphological differences include egg developmental morphology (e.g., size and shape of egg [30], composition of dorsal respiratory appendages in the egg chamber [31,32]), and embryonic developmental timing. Additionally, each lineage has experienced divergent genomic evolution as a result of differences in mutational processes. For instance, differences have been documented in insertion and deletion rates [33–35], as well as specific chromosomal inversions and transposition events [29]. Thus, *Drosophila* provides a powerful model system for studying how developmental suites of genes still manage to produce the basic body plan of a fly despite divergent processes affecting embryogenesis and genome composition.

In this study, we show how a class of equivalent developmental CRMs track evolutionary change in different *Drosophila* lineages. These CRMs act as neurogenic ectoderm enhancers (NEEs) and function to drive gene expression in the early embryonic neuroectoderm before gastrulation has commenced [36]. The NEEs map to unrelated loci: the *rhomboid* (*rho*) locus, which encodes a serine protease; the *vein* (*vn*) locus, which encodes an epidermal growth factor receptor ligand; the *ventral neurons defective* (*vnd*) locus, which encodes an NK-2 class homeobox transcription factor; and the *brinker* (*brk*) locus, which encodes a dipteran-specific helix-turn-helix repressor. The NEEs from these loci are located variably in either upstream or intronic positions and do not share sequence homology indicative of a common evolutionary origin.

Each NEE has independently evolved an organized cluster of common binding sites defined by three sequence signatures in D. melanogaster [36]. First, there are one to two pairs of a Dorsal binding sites closely juxtaposed (<20 bp) to a CA-core E-box motif, which is variably bound in different cells by either Twist basic helix-loop-helix (bHLH) complexes or the Snail C_2_H_2_ zinc-finger repressor [37]. Synergistic activation by Dorsal and Twist at specific positions along the Dorsal morphogen concentration gradient has been well documented [38–40]. Second, there is a unidirectionally oriented site, the μ motif, which is situated at a relatively fixed distance from the Dorsal–Twist pair, and which resembles the binding site for another co-activator, Dorsal interacting protein-3 (Dip3) [41–43]. Third, there is a unidirectionally oriented site, which is a composition of overlapping binding sites for the Notch signaling effector Suppressor of Hairless [Su(H)] and Dorsal. Other nuclear factors may also operate at NEE motifs in distinct territories along the dorsal/ventral (D/V) axis [44]. These diverse motifs co-occur in a 240–320 bp window defining each NEE [36]. Here, we show that selection acts on the organization of NEE binding sites to fine-tune the threshold readouts along the Dorsal concentration gradient.

## Results

### Identification of Multiple Drosophilid NEEs

**Figure 1:**
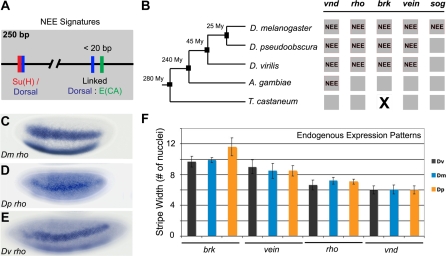
Identification and Characterization of NEE-Driven Loci in Diverse Drosophilid Genomes (A) NEE sequences in many *Drosophila* genomes share a bipartite Su(H)/Dorsal motif and one or two pairs of linked Dorsal and CA-core E-box [E(CA)] motifs. (B) NEE sequences are found at diverse and unrelated loci in dipteran genomes. Other species shown are Anopheles gambiae and Tribolium castaneum. Gray boxes indicate loci; X indicates that a locus is not found; NEE indicates that the locus contains an NEE. My, million years. (C–E) Endogenous expression of the NEE-bearing *rho* loci is depicted for D. melanogaster (C), D. pseudoobscura (D), and D. virilis (E) stage 5 embryos. All images are lateral views of embryos with anterior pole to the left and dorsal side up. (F) Measurements of the width of lateral stripe of expression in number of nuclei at 50% egg-length at relative stage 5 cellularizing embryos is shown for four NEE-bearing loci in all three species.

**Figure 2:**
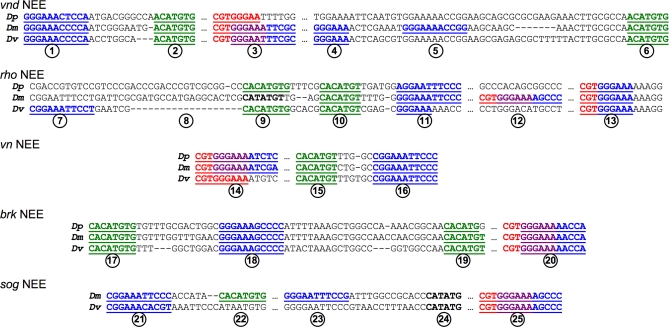
Configurations of D. melanogaster, D. pseudoobscura, and D. virilis NEE Sequences Sequences of NEE *cis*-elements described in this study were aligned from the *vnd*, *rho*, *vn*, *brk*, and *sog* loci from D. pseudoobscura (top aligned sequence), D. melanogaster (middle aligned sequence), and D. virilis sequences (bottom aligned sequence). Particular details (circled numbers) are discussed in the text. Dorsal motifs are shown in blue, Twist CA-core E-boxes are depicted in green, and Su(H) motifs are depicted in red. Overlap between Dorsal and Su(H) motifs are depicted in purple. Only the regions containing these motifs are shown. Ellipses (“…”) indicate intervening sequences that are not shown. See Table S1 for the full-length sequence.

We identified NEE-type sequences across the D. melanogaster, D. pseudoobscura, and D. virilis genomes in order to determine how a set of coordinately regulated gene loci co-evolve in a given lineage. We found that the NEE signatures of paired Dorsal–Twist binding sites (5′-SGGAAADYCSS and 5′-CACATGT, respectively) and a Su(H) site overlapping a separate Dorsal site (5′-CGTGGGAAAWDCSM, Su(H) site underlined) were present together in a single CRM across many loci (Figures 1A, 1B, and 2). We refer to such loci as “NEE-bearing” genes. Interestingly, the D. melanogaster NEE signature of an oriented and positioned μ motif (5′-CTGRCCBKSMM) was not discernable in enhancers from either the D. virilis or the D. pseudoobscura genomes.

From these three genomes, we cloned and assayed in transgenic stage 5(2) D. melanogaster embryos all NEE sequences from these species, which comprised five NEEs from D. melanogaster, five NEEs from D. virilis, and four NEEs from D. pseudoobscura, making a total of 14 distinct NEE-like sequences (Figure 1 and Table S1). These sequences include new NEE-like sequences at the *short gastrulation* (*sog*) loci (Figures S2 and S3). All of these sequences had interesting lineage-specific properties, described below.

To verify the endogenous expression patterns of NEE-bearing genes in these three species, we performed whole-mount antisense RNA in situ hybridization experiments using species-specific probes. Because the developmental timing of embryogenesis differs in the different species, we focused on one developmental time point corresponding to embryonic stage 5(2), when the embryo is midway through cellularization (Figure S1). At this point, the cell walls are 50% elongated and are easily identifiable under bright field microscopy. For D. melanogaster embryos growing at 25 °C this corresponds to ∼2 h 45 min after egg deposition. For D. virilis embryos growing at 25 °C, stage 5(2) corresponds to ∼5 h after egg deposition (Figure S1). However, in all three systems, NEE-driven reporters show earlier activity in late stage 4. This early pattern of activity in late stage 4 sometimes includes faint staining in the mesoderm that disappears by stage 5(2), at which point the lateral stripes are at their most robust and most reproducible levels.

We found that the expression patterns of orthologous genes across different species were significantly more alike than the expression patterns of NEE-bearing genes within the same species (Figure 1C–1F), despite differences in developmental timing, egg size, and genomic content (Table 1). For example, staining with species-specific *rho* probes reveals similar lateral stripes of expression in all three species (Figure 1C–1E). The span of expression for all NEE-bearing genes was quantitatively similar in terms of the number of nuclei along the D/V axis at 50% egg-length at the same embryonic stage. Similar observations were obtained at 25% and 75% egg-length (unpublished data). The differences in spans of expression are determined by the dorsal border of expression, as shown by the fact that the equivalent ventral mesodermal region remains unstained in each species. For example, the *vnd* genes across all three species were expressed in a narrow lateral stripe in the ventral neurogenic ectoderm spanning about six nuclei, whereas the *brk* genes were expressed more broadly in a domain spanning 10–12 nuclei, including more dorsal nuclei than the *vnd* expression pattern (Figure 1F).

**Table 1:**
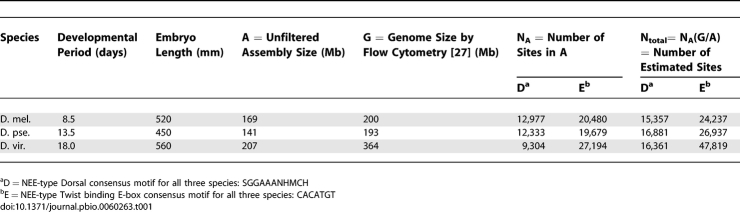
Differences Among D. melanogaster, D. pseudoobscura, and D. virilis

### Rapid Divergence of NEE Sequences

Despite the similar patterns of endogenous expression between NEE-bearing orthologs, the amount of sequence divergence among these 14 enhancers is such that no two orthologous NEEs are more than ∼60% identical as a result of numerous substitutions, insertions, and deletions. In some cases, the amount of identity between orthologous enhancers is as low as ∼44%. This sequence divergence could represent both neutral drift processes and/or positive selection operating in each lineage.

Although these NEE sequences share certain signatures, some of these are undoubtedly examples of stabilizing selection creating de novo sites that compensate for sites lost by mutation. We find that this has occurred for all types of NEE motifs. For example, overlapping Su(H)/Dorsal motifs are not always present in the same location in some species for the *vnd* and *rho* NEEs (details 3 and 12 in Figure 2). Also, entirely new paired Dorsal and CA-core E-box motifs are found in the *rho* and *sog* NEEs (details 7–9, 11, 22, and 23 in Figure 2). In the *rho* example, the spacing has also been adjusted either through substantial deletions in the D. virilis lineage, or else through insertions in the D. melanogaster lineage (detail 8 in Figure 2). Thus, previously reported examples of stabilizing selection [26,45] appear to represent a general property of the NEE equivalence class of enhancers.

Not all NEE-bearing loci in one species are necessarily NEE-bearing in other species. We have found that the D/V patterning gene *sog*, which encodes a chordin-like inhibitor of the BMP/*dpp* signaling pathway, is an NEE-bearing locus in the D. melanogaster lineage but not in the D. pseudoobscura and D. virilis lineages (Figures S2 and S3). Previous work identified an intronic lateral stripe enhancer in the D. melanogaster locus (LSE in Figure S2E) [46]. This enhancer was identified by its cluster of multiple Dorsal binding site but lacks Su(H), E-box and μ motifs. We find that this intronic enhancer is not as well conserved across species as the upstream NEE-like sequences. Moreover, the *D. melanogaster sog* NEE drives the broadest lateral stripe of expression of all the other NEEs we have tested, spanning 15 nuclei across the entire embryo (see Figure 1F). This *sog* NEE recapitulates the endogenous expression pattern (Figure S2A and S2B). We also tested the orthologous upstream sequence from D. virilis and found that it, too, recapitulates its endogenous expression pattern (Figure S2C and S2D). These upstream NEE-like sequences contain Dorsal-linked TA-core E-boxes (5′-CATATG) and bipartite Su(H)/Dorsal motifs in all three species we studied, but they do not always contain the paired Dorsal and CA-core E-box sites, which are unique to the *D. melanogaster sog* NEE (Figure S3). In addition, when we tested the poorly conserved D. virilis sequence orthologous to the intronic lateral enhancer in transgenic D. melanogaster embryos, we observed only weak, patchy staining (unpublished data). Overall, these results show dynamic evolutionary history across individual CRMs, gene loci, and genomes.

### What: Lineage-Specific Thresholds for Morphogen Gradients

An unknown portion of the substitution, insertion, and deletion mutations observed in the NEE *cis* sequences may be lineage-specific adaptations that stabilize changes occurring in *trans*. To address this question, we assayed individual enhancers from all three species in transgenic stage 5(2) D. melanogaster embryos, with multiple lines per enhancer to ensure reproducibility. This assay effectively decouples lineage-specific changes in *trans* from changes in *cis* by testing all enhancers in the same *trans*-environment of D. melanogaster. If these enhancers have evolved to compensate for lineage-specific changes in the *trans* regulatory environment, then we should observe similar directional changes for the entire equivalence class from one lineage when tested in transgenic D. melanogaster embryos.

**Figure 3:**
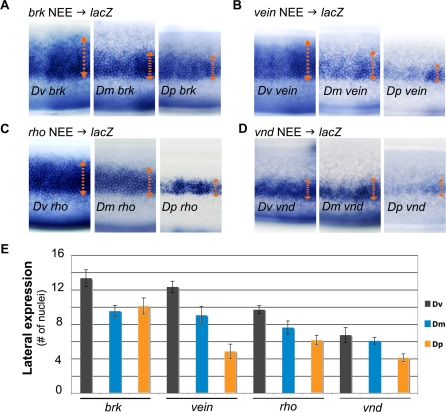
Analysis of NEE Activities in Transgenic D. melanogaster Embryos (A–D) NEE-driven *lacZ* transgenes assayed in D. melanogaster embryos from the *brk* (A), *vn* (B), *rho* (C), and *vnd* (D) loci demonstrate that D. virilis enhancers tend to drive broader stripes than D. melanogaster enhancers. Similarly, D. melanogaster enhancers tend to drive broader stripes than D. pseudoobscura enhancers. (E) Quantification of the widths of lateral stripes of expression at 50% egg length across multiple embryos from several lines supports this general trend. The in situ staining experiments in this figure were conducted in parallel and with the same anti-sense *lacZ* probe to facilitate comparisons.

**Figure 4:**
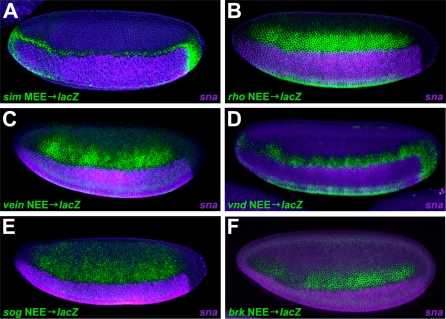
Determination of Dorsal/Ventral Borders of Expression of NEE-Driven Transgenes Fluorescent double-labeling in situ hybridization experiments were carried out using probes for *snail*, which marks the mesoderm (purple), and *lacZ*, which is driven by the indicated enhancers in the lateral regions of the embryo (green). All enhancers span D/V expression domains that abut the sharp *snail* border in the mesoderm and continue to more dorsal nuclei. (A) *D. melanogaster sim* mesectodermal enhancer (MEE) driving *lacZ* shows that NEE driven *lacZ* expression (B–F) enters the mesectoderm because both NEE and MEE activities equally abut the mesodermal border*.* (B) *D. melanogaster rho* NEE driving *lacZ.* (C) *D. melanogaster vein* NEE driving *lacZ.* (D) *D. melanogaster vnd* NEE driving *lacZ.* (E) *D. melanogaster sog* NEE driving *lacZ.* (F) *D. melanogaster brk* NEE driving *lacZ.*

**Figure 5:**
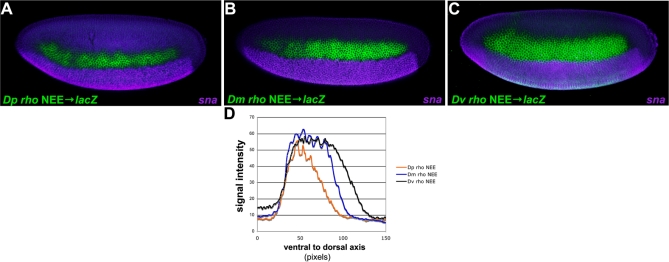
Fluorescent Double-Labeling of *rho* NEE-Driven Transgenes (A–C) Double-staining (*lacZ* and *snail* expression in green and purple, respectively) for *rho* NEE driven transgenes from D. pseudoobscura (A), D. melanogaster (B), and D. virilis (C) and *snail* (*sna*) expression in stage 5 embryos. The sharp *snail* border of expression provides a landmark to align expression patterns across embryos. (D) All three enhancers drive expression patterns of similar intensity although the D/V axis although the width of the stripe is narrower for D. pseudoobscura, and wider for D. virilis than the D. melanogaster NEE driven transgene.

Interestingly, despite similar functional outputs of orthologous NEEs in the context of their native genomes (Figure 1F), the NEEs from each species have unidirectionally modified activities in transgenic D. melanogaster embryos when compared with all the NEEs as a group from other species (Figure 3; in situ detection experiments were conducted in parallel). Specifically, the D. virilis enhancers consistently drive expression of reporters in a significantly more robust and expansive lateral stripe than the D. melanogaster enhancers in transgenic D. melanogaster stage 5(2) embryos, whereas D. pseudoobscura enhancers drive expression in a narrower stripes than the D. melanogaster enhancers (Figure 3). We have also verified this by conducting fluorescent double-label in situ hybridization of NEE-driven *lacZ* reporter lines using anti-*lacZ* and anti-*snail* RNA probes; *snail* labels the mesoderm (Figures 4 and 5). These experiments reveal that NEE-driven *lacZ* expression immediately abuts the mesodermal border without overlapping with it, which is consistent with ventral repression of NEEs by Snail.

The *vnd* enhancers, which produce the narrowest stripes of the NEE modules tested, showed the smallest differences in relative expression patterns when assayed in D. melanogaster (Figure 3D and 3E). It is possible that any extra activation potential or the need for it in *vnd* NEEs is masked by mechanisms that set the more restricted dorsal limit of expression, such as repression by the Ind and Msh homeodomain proteins or Schnurri-mediated repression via BMP signaling [47–50].

Similar results across NEE orthologs were obtained when we measured *lacZ* transcript intensity levels along the D/V axis using confocal microscopy. By aligning the sharp border of *snail* expression we see similar differences in stripe width (Figure 5). These results also show that, in many cases, it is the width of the stripe and not its intensity that changes between enhancers (Figure 5D). This suggests that the changes occurring in *cis* specifically affect the morphogen concentration thresholds that are being sensed by these enhancers.

### How: Precise Organization Controls NEE Function

As the D/V patterning system is known to be mediated primarily by Dorsal and Twist proteins, we decided to investigate the configuration of their binding sites in all of the enhancers and relate these in turn to their widths of expression across the lateral regions of the embryo. We found, first, with a few exceptions, that the CA-core E-box motif 5′-CACATGT is remarkably constant across the NEE sequences of all three species. Second, the Dorsal site occasionally sustains some point mutations. Third, there appear to have been many insertions and deletions that have adjusted the spacing between these two sites. Thus, the changes from all three types of variables (Twist sites, Dorsal sites, and their spacing) have served to alter the spacing in most cases, and occasionally to alter the number and quality of paired Dorsal and Twist sites (see Figure 2). We therefore suspected that the different widths of expression correlated with just these variables, as predicted by quantitative modeling [51]. In this manner, lineage-specific threshold readouts would be consistent with stabilizing selection in *cis* for diverse changes occurring in *trans*.

To test this hypothesis of genome-wide threshold adaptations, we decided to alter specific NEE sequences that differed only slightly in their Dorsal and Twist binding motif configuration relative to another NEE sequence that nonetheless differed greatly in the span of expression along the D/V axis. Of relevance, we also noted that the broadest NEE transgenes had a spacing between the Dorsal site and the adjacent E-box close to 7–12 bp (compare Figure 4 with sequences in Figure 2). Excepting the *vnd* NEEs, which may be constitutive targets of dorsally expressed repressors [47–50], most NEEs have increasingly narrow stripes the farther they are from this optimal spacer.

**Figure 6:**
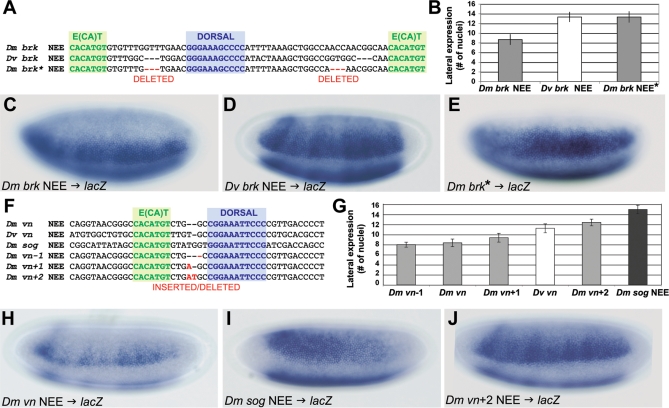
Precise Changes in NEE Organization Determine Lineage-Specific Threshold Readouts of Morphogen Gradient (A–E) Minimal modification of the *D. melanogaster brk* NEE configuration so that it resembles the D. virilis spacing (A) is sufficient to expand expression to levels seen for the *D. virilis brk* NEE-driven transgene (B–E). Asterisk (*) indicates that the spacing has been mutated in an otherwise wild-type *D. melanogaster brk* NEE. (F–J) A series of minimal modifications to the *D. melanogaster vein* (*vn*) NEE configuration (F) so that it differs by −1 bp, 0 bp (wild-type), +1 bp, and +2 bp, which is similar to the broadly expressed *D. melanogaster sog* NEE configuration, yields a series of monotonically increasing widths for lateral stripes of expression (G–J). The in situ staining experiments in this figure were conducted in parallel and with the same anti-sense *lacZ* probe to facilitate comparisons.

**Figure 7:**
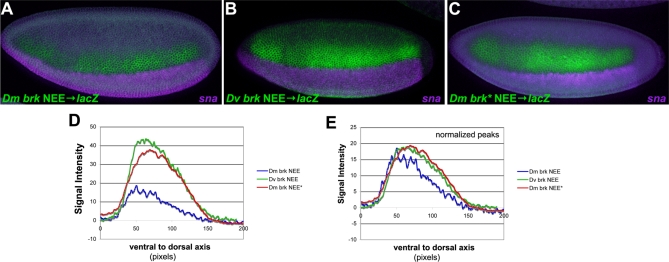
Fluorescent Double-Labeling of *brk* NEE Driven Transgenes (A–L) Double-staining (*lacZ* and *snail* expression in green and purple, respectively) for *brk* wild-type and mutated enhancers from D. melanogaster and D. virilis. (A) *D. melanogaster brk* NEE driving *lacZ*. (B) *D. virilis brk* NEE driving *lacZ*. (C) *D. melanogaster brk* NEE deletion mutant (*) driving *lacZ* (as in Figure 4) is depicted. The spacing between both pairs of Dorsal and Twist sites has been adjusted to resemble *D. virilis brk* NEE spacing (see Figure 6A). (D) The *D. melanogaster brk* enhancer with adjusted Dorsal–Twist spacing drives *lacZ* expression over a similar width and at similar intensities to the *D. virilis brk* enhancer. For a wide-range of signal intensity thresholds, the width of the stripe is greater in these enhancers with optimal spacers is much greater than the wild-type *D. melanogaster brk* NEE. (E) The same data as in D normalized to peak intensity shows that there is still a measurable difference in stripe width in embryos carrying enhancers with optimal *brk* Dorsal–Twist configuration.

For example, the Drosophila brk NEEs have a conserved organization consisting of a central invariant Dorsal site flanked on either side by invariant CA-core E-box motifs (5′-CACATGT) (Figure 2 details 17–19, and Figure 6A). However, the D. virilis Dorsal to E-box spacer is shorter by exactly 3 bp on either side of the Dorsal motif relative to the D. melanogaster NEE (Figures 2 and 6A), in addition to many other substitutions and insertions and deletions (indels) throughout these enhancers. Recall that while the *D. melanogaster brk* NEE drives a lateral stripe of about eight or nine nuclei wide, the *D. virilis brk* NEE drives a lateral stripe of ∼13 nuclei in D. melanogaster stage 5(2) embryos (Figure 6B–6D). We therefore reduced the D. melanogaster NEE Dorsal site to E-box spacers by 3 bp on each side, mimicking the D. virilis configuration. This precise adjustment in spacing is sufficient to broaden the expression of the *D. melanogaster brk* NEE driven transgene to *D. virilis brk* NEE levels (Figure 6B and 6E). These in situ detection experiments were conducted in parallel to aid comparison. Furthermore, double labeling with probes to the mesodermal marker *snail* and the *lacZ* transgene shows that this functional change extends to both intensity of expression as well as expansion of the dorsal border of expression (Figure 7). However, even after normalizing the peak concentrations, a measurable difference in width is still evident (compare Figure 7D and 7E). These in situ detection experiments were also conducted in parallel to aid comparison.

In a similar example, the Drosophila melanogaster vn and *sog* NEEs possess the same Dorsal motif, which otherwise tends to vary at other loci (Figure 6F). This Dorsal motif (5′-CGGAAATTCCC) in each enhancer is situated 4 bp and 6 bp from the E-box motif (5′-CACATGTG) in the *vn* and *sog* NEEs, respectively (Figure 6F). Yet despite this similar NEE configuration in an otherwise nonhomologous DNA sequence, the *sog* NEE drives a broad lateral stripe of expression (∼15 nuclei; Figure 6G–6I) that is almost twice as broad as the *vn* enhancer (about eight nuclei; Figure 6G and 6H). Interestingly, the *D. virilis vn* NEE has an intermediate spacer of 5 bp and drives a lateral stripe of expression of intermediate width (∼11 nuclei; Figure 6G). We then compared a series of modified *D. melanogaster vn* NEE-driven transgenes possessing spacers adjusted by −1 bp, 0 bp (i.e., wild-type), +1 bp, and up to +2 bp, which mimics the *sog* NEE spacer, and found a monotonically increasing width in the lateral stripe of expression (Figure 6G–6J; in situ detection experiments conducted in parallel).

Thus, both the natural range of NEE configurations within each genome and across all three genomes, together with our functional manipulation of NEE configurations, confirm that the Dorsal site/E-box configuration controls the precise extent of D/V expression by extending the dorsal border of expression, where concentration of key activators is limiting. Thus, not only is there is an optimal organization for maximum affinity, but there is also a range of affinities that is exploited by natural selection to maintain precise threshold readouts of the concentration gradient of a developmental morphogen.

### Why: Lineage-Specific Changes in the D/V Patterning System

We next investigated diverse possibilities for lineage-specific selective pressures that might have caused NEEs across each genome to functionally adapt in similar directions via changes in spacing between the binding sites of cooperative activators. Such reasons might help explain why D. pseudoobscura NEEs have the weakest and narrowest stripes of expression in D. melanogaster embryos, while D. virilis NEEs have the strongest and widest stripes of expression when NEEs from all three species are tested in D. melanogaster embryos (Figure S8).

First, amino acid substitutions have occurred in the known NEE transactivators Dorsal and Twist (Figure S4, and unpublished data). Some enhancer evolution could therefore conceivably be due to stabilizing selection for changes in the *trans* factors themselves. Relative to the D. melanogaster Dorsal peptide sequence, these changes include a few non-synonymous substitutions in the DNA-binding REL homology domain (RHD, underlined sequence in Figure S4), as well as several non-synonymous substitutions and peptide indels in the non-DNA-binding regions. Interestingly, some of these amino acid substitutions in the DNA-binding domain correspond to known mutations that either reduce or augment Dorsal–Twist synergistic activation [52]. A lysine (K) to leucine (L) change in the D. pseudoobscura Dorsal RHD corresponds to a position that augments activation when mutated to alanine (A) in D. melanogaster Dorsal (see M7 in Figure S4). Both are changes of a basic side-chain to an aliphatic one. Such changes in D. pseudoobscura Dorsal might allow the evolution of weaker target NEEs. Remarkably, another mutation in the D. virilis Dorsal RHD corresponds to a position that reduces activation when mutated in D. melanogaster Dorsal (see M23 in Figure S4). Such a change in Dorsal might necessitate the evolution of stronger D. virilis NEEs. Future studies will investigate Dorsal protein and NEE co-evolution.

A second potential reason for genome-wide adaptations could also be changes in the protein expression levels. Staining with polyclonal antibodies made to D. melanogaster Dorsal and Twist factors reveals ventral to dorsal nuclear concentration gradients in all three species, with detectably slightly narrower nuclear Dorsal concentration gradients in D. virilis and broader nuclear Dorsal concentration gradients in D. pseudoobscura relative to the D. melanogaster gradients (Figures S5 and S6). The ratio of intensities for dorsal cytoplasmic levels of Dorsal antigen versus ventral nuclear levels are qualitatively similar across species and indicate that the shapes or profiles of the nuclear concentration gradient are comparable, even though the absolute intensities may not be comparable (Figure S7). Nonetheless, if the Dorsal morphogen gradient really is augmented in the smaller D. pseudoobscura embryos, and reduced in the larger D. virilis embryos, such changes would be consistent with the NEEs adapting to lineage-specific concentration readouts (Figure S8).

We also see a third potential reason for lineage-specific threshold readouts related to genome evolution (Table 1). Flow cytometry analyses of *Drosophila* genome sizes have confirmed a diverse range of sizes from 130 Mb for D. mojavensis up to 364 Mb for D. virilis [27]. D. melanogaster and D. pseudoobscura have intermediate genome sizes of 200 Mb and 193 Mb, respectively [27]. Of interest, we find that the total number *N*_D_ = *N_A_G*/*A* of estimated NEE-style Dorsal motifs (5′-SGGAAABYCCH), where *N*_A_ is the number of motifs found in the unfiltered assembly of size *A* in a genome of size *G*, is relatively constant across all three genomes, the total number *N*_E_ of NEE-style CA-core E-boxes (5′-CACATGT) is 2-fold greater in D. virilis than in D. melanogaster (Table 1). We also note that the relative increase of this motif is a secondary trend related to a simple expansion (D. virilis) or compaction (D. melanogaster) of 5′-CACA repeats occurring primarily in euchromatic regions of the genome (unpublished data). In general, longer microsatellites have been documented in D. virilis than in D. melanogaster [35]. However, a potential effect of a 2-fold greater number of genomic 5′-CACATGT motifs might be to reduce the effective free nuclear concentration gradient of Twist bHLH complexes in D. virilis relative to D. melanogaster via background sequence sequestration [53]. Such an effect would also be consistent with the observed adaptive trend to a lower concentration threshold readout in D. virilis than in D. melanogaster (Figure S8B). Thus there are potentially several lineage-specific changes affecting the activity or profile of the dorsal/ventral morphogen system that would necessitate the observed genome-wide adaptations in downstream target enhancers.

## Discussion

In this study we demonstrated the effectiveness of studying gene regulatory evolution in the context of a CRM equivalence class consisting of all genomic sequences that regulate nearly identical patterns of activity by interacting with a common set of transcription factors.

Equivalence classes of CRMs represent molecular examples of parallelisms, which can be defined as similar, homoplastic patterns of evolutionary innovation constrained by a restrictive set of available regulatory mechanisms [54]. For several important reasons, generalization of CRM logic for a class of functionally and mechanistically equivalent CRMs is difficult to obtain from the phylogenetic study of a single CRM. First, insufficient time for divergence away from an ancestral CRM can leave much superficial similarity among orthologous CRMs. Second, the persistence of initial organizational constraints present in the ancestral CRM can obscure possible alternative configurations of the class-defining *cis* elements. Third, novel lineage-specific evolutionary adaptations will impede any method that relies purely on phylogenetic conservation.

We used the equivalence class of NEEs from several species to show that selection acts on the organization of enhancers to fine-tune their output. In this case, NEEs have evolved in parallel to adapt to changes in a developmental morphogen gradient, whose activity levels have shifted in different directions in different lineages, necessitating compensatory changes in the *cis* components of downstream targets (Figure S8). Such stabilizing selection, encoded in the configuration of Dorsal binding sites and Twist-binding CA-core E-boxes, reflects species-specific thresholds of activation (compare θ_Dm_, θ_Dp_, and θ_Dv_ in Figure S8). Thus, the D. pseudoobscura enhancers have evolved to respond to higher morphogen concentrations than those in D. melanogaster (θ_Dp_ > θ_Dm_), while the D. virilis enhancers have evolved to respond to lower concentrations than those in D. melanogaster (θ_Dv_ < θ_Dm_). This stabilizing selection is revealed only when finely tuned NEEs are tested in the exogenous concentration gradient of a different species, in this case the reference species *D. melanogaster*.

It should not be surprising that small changes in the linkage between the Dorsal and Twist complex binding sites could have such a dramatic effect on the D/V range of expression. First, evolutionary changes in the dorsal border of neuroectodermal gene expression are a simple readout of the most limiting amount of nuclear Dorsal, further limited by limiting amounts of Dorsal target proteins working as Dorsal co-factors, such as Twist bHLH complexes [39,40,55]. Second, recent studies on the Bicoid morphogen gradient, which simultaneously patterns the anterior/posterior axis, suggest that its precision is pushed to the physical limits imposed by the stochasticity of molecules [56–58]. Therefore, precision in the morphogen gradients patterning the embryonic axes should indicate a precision in CRM readouts, as we have shown here for one class of D/V enhancers and others have indicated for diverse anterior/posterior enhancers [59,60].

It should also not be surprising that it is the site linkage that is adjusted by natural selection rather than the quality of the binding sites. Quantitative modeling of the classical Dorsal morphogen system has placed heavy emphasis on the quality of binding sites as determinants of differential threshold readouts by target enhancers in the mesoderm, mesectoderm, and neuroectoderm [51]. However, we do not believe this is entirely at odds with our results showing evolutionary modification of NEE activity via organizational linkage because it may indicate that within the neuroectodermal territory, where Dorsal protein is present in limiting amounts, binding sites are likely to have already evolved to be high affinity sites. This would indicate that in this embryonic territory, extra affinity can only be achieved by optimizing positional linkage between these cooperatively binding factors. This in turn implies that these same factors have relatively fixed steric dimensions.

Another potential locus and/or cause of selection lies in the protein-coding sequences of the morphogens themselves, as has been shown for other *trans* factors [61,62]. Both Dorsal and Twist are used as combinatorial inputs for other regulons in other tissues (e.g., mesoderm and mesectoderm) and the pleiotropic consequences of changes to either protein–protein interaction motifs or DNA-binding domains might limit the number of possibilities for such changes. Additionally, such protein-coding changes may not effectively or precisely target the Dorsal–Twist interactions where their amounts are limiting and/or affect the interaction in the continuously graded fashion that we have documented. Nevertheless, stabilizing selection for changes in the Dorsal peptide sequence itself or other *trans* factors could be explored by future *trans* complementation assays. However, in many cases, it may be difficult to disentangle evolutionary cause and effect between co-evolving loci throughout the genome because sequence changes may be either the initiating causes or the products of selection for developmental homeostasis.

In summary, the coordinate changes in both sequence and activity shown by the neuroectodermal enhancers from each species provide strong evidence for functionally adaptive *cis*-regulatory evolution. The number of fixed nucleotide changes corresponding to these parallel molecular adaptations occurring across a genome is minimal, and corresponds to a few indels between Dorsal and Twist sites across the NEEs in a single genome. Occasionally, new Dorsal and Twist sites with optimal spacing are presumed to have been selected in individual species for the *vnd*, *rho*, and *sog* NEEs while in others only the spacing has changed between existing sites. Our results show that natural selection can act with ease to fine-tune CRM organization and thus calibrate enhancer activity over a wide dynamic range. As such, CRM organization may represent a large and unexplored locus of stored adaptive information.

## Materials and Methods

### Fly stocks.

D. melanogaster strain w1118 was used for *P*-element transformations of all reporter constructs. D. virilis and D. pseudoobscura were obtained from the Tucson *Drosophila* Stock Center.

### Cloning and in situ hybridization.

D. melanogaster, D. virilis, and D. pseudoobscura embryos were collected, and subsequently fixed. Hybridization with digoxigenin-labeled antisense RNA probes was conducted as previously described [63]. Fluorescent multiplex in situ hybridization methods were performed as previously described [64]. Briefly, primary antibodies were used to detect fluorescein isothiocyanate- and digoxigenin-labeled antisense RNA probes (used 1:400, Invitrogen), followed by detection of primary antibodies using secondary antibodies labeled with Alexa Fluor dyes (used 1:500, Invitrogen). Images of fluorescently labeled embryos were acquired on a Nikon Eclipse 80i scanning confocal microscope with a 20× objective lens. Sum projections of confocal stacks were assembled and plot profiles of the RNA transcripts were analyzed using the ImageJ software. Anti-sense endogenous probes were created by PCR amplification from genomic DNA with a T7 RNA polymerase promoter included on the reverse primer (see Supplemental methods for all primer pairs). DNA fragments for injection were cloned into the [-42EvelacZ]-pCaSpeR vector and introduced into the D. melanogaster as described previously [3]. Between three and seven independent transgenic lines were obtained for each construct: Dm brk NEE (657 bp), Dp brk NEE (859 bp), Dv brk NEE (744 bp), Dm rho NEE (871 bp), Dp rho NEE (843 bp), Dv rho NEE (726 bp), Dm vn NEE (919 bp), Dp vn NEE (858 bp), Dv vn NEE (836 bp), Dm vnd NEE (1020 bp), Dp vnd NEE (1305 bp), Dv vnd NEE (1093 bp), Dm sog NEE (550 bp), and Dv sog NEE (871 bp).

### Immunochemistry.

For protein expression experiments, *Drosophila* embryos for all species were fixed in 3.7% formaldehyde for 20 min at room temperature. Polyclonal rabbit anti-Dorsal, guinea pig anti-Dorsal, and rabbit anti-Twist antibodies were used for primary detection. Secondary anti-rabbit antibodies conjugated to FITC (Roche Applied Sciences) and anti-guinea pig conjugated to TRITC (Sigma-Aldrich) were used for visualization.

### Bioinformatics.

Whole-genome scans for sequences matching enhancer models were conducted using multiple techniques to verify results. These methods included searching fly genomes using scripts written in PYTHON and as well as local-alignment-based methods, primarily the VISTA suite of whole genome alignments. Dialign2 was used for additional sequence alignment and to determine nucleic acid identity.

### DNA construct cloning and injection.

The enhancer sequences used in this study have been deposited in GenBank (http://www.ncbi.nlm.nih.gov/Genbank/index.html) with accession numbers FJ169871–FJ169884. D. melanogaster and D. virilis DNA fragments containing identified enhancer elements were amplified from genomic DNA with the following primer pairs (lowercase letters denote flanking primer sequence used to introduce the restriction site indicated in brackets): Dm rho: [BsaI] ccgcataattcgagaccCAGTTAAGTGAGTCGCTTTCAGG, ccgcataattcgagaccTAGATAGATATACCCATCCTGGCC; Dv rho: [BsaI] ccgcataattcgagaccCTGGGAAGTTGCACGAGAGACGC, ccgcataattcgagaccGAGAAACTCTTCTGGCACAACGC; Dp rho: [EcoRI] ccgcggaattcACTAGTGGAAGCTGCTCTACAGACGCG, ccgcggaattcACTAGTCACACACAGCGAAGCACTGAGA; Dm vnd: [EcoRI] ccgcggaattcGGAAGATTGGGCGTTGAAAGC, ccgcggaattcCGGCCATTCACACGATTGACACA; Dv vnd: [Mef1] ccgcgcaattgCTGTTTGGCCTGGCTGGC, ccgcgcaattgATGGCCGGAAAGCAACACAATGG; Dp vnd: [BsaI] ccgcataattcgagaccGTATTTCGAAGATGCATTTGTTTGC, ccgcataattcgagaccTGAATGGCCGGAAGGTCCAACAAG; Dm vn: [BsaI] ccgcataattcgagaccCAGTTCTGGATCTTCCGAATCACC, ccgcataattcgagaccAAATTTGTAGCCAGCGGCGACG; Dv vn: [EcoRI] ccgcggaattcCATATGTTGCCCCTTTGCTGTTGC, ccgcataattcgagaccAAATTTGTAGCCAGCGGCGACG; Dp vn: [BsaI] ccgcataattcgagaccTGTGGGGCAATATTTTCTTTTTAGC, ccgcataattcgagaccCTAAAAATGCAACTCCAACTTGTCTG; Dm brk: [EcoRI] ccgcggaattcTTGGTCGGAAAATACCTGCGC, ccgcggaattcATTGTGTGGCGTTAGAAAGATATGG; Dv brk: [EcoRI] ccgcggaattcTGTCCGGGCTTATGGATCG, ccgcggaattcTGCATTATCCGTGCTAAGTTTGGG; Dp brk: ccgcTCTAGAAAAATGCCGAACAGGTACGTCG, ccgcTCTAGAAAATCATATCCTAACCCCATCTGGG; Dm sog NEE: [EcoRI] ccgccgaattcTGTTTATGGCAGCCAATTGATGCCGA, ccgccgaattcgatgatctagaatcgcacggagag. The *brk* and *rho* enhancers were mutagenized using overlap extension PCR with the following primers: Dm brk D/T1 (letters flanking deletion underlined): GGCACAGGCACACATGTGTGTTTGTGAACGGGAAAGCCCCATTTT; Dm brk D/T2 (letters flanking deletion underlined): GCCCCATTTTAAAGCTGGCCCAACGGCAACACATGTTCATGTTAG; Dm vn-1 (letters flanking deletion underlined): GGACAGGTAACGGGCCACATGTCTGCCGGAAATTCCCCGTTGACCCCTG; Dm vn+1 (inserted letter underlined): GGACAGGTAACGGGCCACATGTCTGAGCCGGAAATTCCCCGTTGACCCCTG; Dm vn+2 (inserted letters underlined): GGACAGGTAACGGGCCACATGTCTGATGCCGGAAATTCCCCGTTGACCCCTG.

### Probes for whole-mount in situ hybridization.

The following primer pairs were used to amplify probes for each of the indicated genes from each species with the T7 promoter sequence indicated): Dm rho: ATCTGGGCTATGCTCTCTACACC, T7-TTAACTGCAAACGGTAACGATAACG; Dv rho: GCCGTCTACACGCAGTACTTCG, T7-CATTTGTTTACACGTTTCGGCCCG; Dp rho: TTGCCATCTTCGCCTACGATCG, T7-GCTTAGGAGACACCCAAGTCG; Dm vn: CTTTGCGGCACCCACCGTTTT, T7-TCCACTCACTATAATTTTCGCTCAC Dv vn: GAGTAGAAGATATATGCGTATGAGC, T7-GTTCACAGCCATTTTAACTGCTTCG; Dp vn: AATTTTGGAGGACGCCATGTATGCG, T7-ACCGATTCCATCACCGAGTGG; Dm brk: GAAATACAACATTCACCGCCGG, T7-TCAGGATTGGCACTTGTATTGGC; Dv brk: ATTTTTGTTACTTCCAAGACGACGG, T7-TATCGAACTTGTTCGCTGTTATCC; Dp brk: CAGCAACCACAGTCCTAACGC, T7-ATCAGGTTGTCGTTGGAGACG; Dm vnd: TCAGTTTGGAATGTGTAGAGTGCGC, T7-GGATAAAAGGCGGCTGGTAGG; Dv vnd: AATGTTTAGAGTGCGGCTGACAACG, T7-TCCAAGGGGGCAGCAATATGG; Dp vnd: TGTCCTACACCTACATCGGTTCC,TCTAGCAGTATTAGGGCCACC; Dm sog: GGAAATGAAGTCCATGTACACCACC, T7-TCTCGTACACCTTGTTGACCACC; Dv sog: GAGGAGATGAAGTCCATGTACACG, T7-CCGTTCTCGTAGATCTTGTTGACG; Dp sog: ATAGTATGTCCCATGCCTCACCG,TTCTGCTCTGGCGAATTTTAAAGC; *Dm cg8117*: ccgccCTAAAAATTGTGCTTTCCGGTTTCG, T7-ATTTACATATTGTGGAAGCCAACGG; *Dm cg8119*: ccgccAACAAGTATCCGACCAACAATCTGG, T7-TCACGTCAGCAGCTTCTTCTCC.

## Supporting Information

Figure S1 Staging of *Drosophila* Embryos (A) The average amount of time to develop to embryonic stages 3, 4, 5, and 6 at 25 °C was plotted for D. melanogaster (blue diamonds) and D. virilis (orange squares). Initial NEE-driven transgene activity is first detectable in mid to late stage 4.
(B) Embryonic stages corresponding to time points measured in (A). Stage 5 can be subdivided into stage 5(1) of incipient cellularization , stage 5(2) of mid-cellularization, and stage 5(3) of recently completed cellularization. These stages can be easily measured by following the leading edge cellularization (yellow arrows). For the purposes of this study activities were compared at stage 5(2), when cellularization is 50% complete.
(847 KB TIF)

Figure S2 Identification of an NEE in the Drosophila sog Locus Endogenous expression patterns of the *sog* loci (E) in D. melanogaster (A) and D. virilis (C) are recapitulated by an upstream NEE-like sequence found at both loci (B, D). A previously described but poorly conserved lateral stripe enhancer (LSE) in the *D. melanogaster sog* intron drives a slightly narrower stripe of expression relative to the upstream NEE. In situ hybridization experiments for two other adjacent genes (*CG8117* and *CG8119*) failed to show lateral stripes of expression in D. melanogaster and are not as well conserved in D. virilis (unpublished data).
(1.89 MB TIF)

Figure S3 Evolution of the Upstream Neuroectodermal Enhancer of the Drosophila sog Locus (A) Comparison of a 2-kb window around the upstream NEE and NEE-like sequences from D. melanogaster (*y*-axis) and D. virilis (*x*-axis) reveals minimal sequence homology in a standard dot plot or graph matrix. Both sequences are functional in D. melanogaster embryos.
(B) More extensive blocks of identity are evident at the Su(H)/Dorsal motif, and at the linked Dorsal and TA-core E-box (5′-CATATG) motifs (red, blue, and green dotted lines, respectively, in (A)).
(C) Conservation of elements in upstream *sog* NEE-like sequence. A separate pair of linked Dorsal and Twist (5′-CACATGT) binding motifs is found at the D. melanogaster sequence (blue and green boxes, respectively) (C).
(471 KB TIF)

Figure S4 Dorsal Peptide Evolution in Divergent Drosophilids Multiple amino acid substitutions (red letters) have occurred in the Dorsal peptide sequence in each of the three lineages examined in this study: D. melanogaster (Dm), D. pseudoobscura (Dp), and D. virilis (Dv). A few of these changes have occurred in the DNA-binding RHD (underlined sequence). A few of these substitutions occur in positions that are known to either diminish (green “class I” mutation) or augment (red “class II” mutation) activation in Dorsal–Twist synergistic activation assays [52]. Others positions in the RHD have had no effects in such assays when mutated (black mutations) or have not been mutated (“?”). Mutation numbers (M7, M9, M21, and M23) refer to their original description [52].
(1.06 MB TIF)

Figure S5 Dorsal Nuclear Concentration Gradient in Divergent Drosophilids Dorsal antibody staining (A, D, and G) and DAPI (4',6-diamidino-2-phenylindole) counter-staining (B, E, and H) in D. melanogaster (A, B, and C), D. pseudoobscura (D, E, and F), and D. virilis (G–I) stage 5 cellularizing embryos. Dissected cross-sections in D. melanogaster (C), D. pseudoobscura (F), and D. virilis (J) embryos show broader D. pseudoobscura (F) and narrower D. virilis (J) nuclear Dorsal concentration gradients than in D. melanogaster (C).
(1.40 MB TIF)

Figure S6 Co-Expression of Dorsal and Twist Proteins in D. melanogaster and D. virilis Embryos Double-staining with Dorsal (A, C, E, and I) and Twist (B, D, F, J) antibodies in stage 5 cellularizing D. melanogaster (A, B, E–H) and D. virilis (C, D, I–L) embryos.
(A–D) Lateral views of double-stained embryos are depicted.
(E–G, I–K) Ventro-lateral views of double-stained embryos are depicted.
(G, K) Merged Dorsal (red) and Twist (green) signals are co-expressed in the yellow nuclei.
(H, L) Bright field views showing cellularizing embryos at comparable stages in the two species.
(3.77 MB TIF)

Figure S7 Cytoplasmic and Nuclear Concentrations of Dorsal Antigen (Close-up of ventral (A, C, and E) and dorsal (B, D, and F) nuclei of early stage 5 D. melanogaster (A, B), D. pseudoobscura (C, D), and D. virilis (E, F) embryos showing transition of signal intensity from the nucleus (thick arrow, ventral surface) to the cytoplasm (thin arrow, dorsal surface).
(2.03 MB TIF)

Figure S8 Model for Lineage-Specific Evolution of Morphogen Threshold Readouts (A) Nuclear concentration gradients for Dorsal for D. melanogaster and D. pseudoobscura embryos.
(B) Nuclear concentration gradients for Dorsal for D. melanogaster and D. virilis embryos.
Lineage-specific changes in the absolute levels of the free Dorsal and Twist nuclear concentration gradients (*y*-axis; solid and dotted curves) necessitated compensatory adaptations at the level of NEE specificity for lineage-specific thresholds (θ) below which NEE activity ceases along the D/V axis (*x*-axis). D. pseudoobscura NEEs are adapted to a higher concentration of Dorsal morphogen (θ_Dp_) in the smaller D. pseudoobscura embryos, such that when they are each tested in transgenic D. melanogaster embryos, they produce narrower stripes of expression (small red arrow) than D. melanogaster genes, which are adapted to θ_Dm_. Conversely, we propose that D. virilis NEEs are adapted to a lower concentration thresholds for Dorsal and Twist morphogens θ_Dv_ as a result of changes in the Dorsal gradient as well as potential genome Twist sequestration effects, such that when they are each tested in transgenic D. melanogaster embryos, they produce broader stripes of expression (large red arrow) than D. melanogaster genes.
(552 KB TIF)

Table S1 List of Enhancer Fragments Used in This Study Binding sites are underlined and in capital letters using the color scheme shown in Figure 2. Primer sequences are shown in capital letters. These sequences have also been deposited with GenBank.
(36 KB DOC)

## Abbreviations

bHLH: basic helix-loop-helix
CRM: *cis*-regulatory module
Dm: Drosophila melanogaster
Dp: Drosophila pseudoobscura
Dv: Drosophila virilis
D/V: dorsal/ventral
indel: insertion or deletion
NEE: neurogenic ectoderm enhancer
RHD: Rel homology domain
Su(H): Suppressor of Hairless
