# Supplementary material for: Evolution Acts on Enhancer Organization to Fine-Tune Gradient Threshold Readouts
Source: PLoS Biol. 2008 Nov 4;6(11):e263. doi: 10.1371/journal.pbio.0060263 (PMC2577699; doi:10.1371/journal.pbio.0060263)
Supplement: Supplementary file 1 [file pbio.0060263.st001.doc]

**Drosophilid NEE sequences**

Crocker, Tamori, Erives (2008)

**>Dm_brk_NEE_657_bp**

TTGGTCGGAAAATACCTGCGCatcctggtggtttatggtgcggccgtaaatgcaagccaagttctttacggcttctctggcacaaaccctaaatgtggattacgctaatattgccccccctaataaaaacggtcgttgtccagggccgaatattgcgtctgat**tggtttttcccacg**attacaattagccggacggacacaaactgacctgagctgacccgcaaaaagacacggttgtccggcagtcggaactgaaggaaactaaaggaaactgagggcaggtcagcgctatggattgtgcactaagttgcttaatccgacgggaaatccaaaacacaacccgagcccgatccttcgctccttcgatttaagccaaagttagaggcacaggc**acacatgtg**tgtttggtttgaac**gggaaagccc**cattttaaagctggccaaccaacggca**acacatgt**tcatgttaggaccgatacaggttgacattccctggaaggatgcacctctgggagattcccacaaccggcagcaggtcatgtccaaccgatcgttgcgggagccacttgtcccgaaaaaatccaaagaaactatcaagtggcgtttagggaaactcaaaactttccaaccacaCCATATCTTTCTAACGCCACACAAT

**>Dp_brk_NEE_859_bp**

GCCGAACAGGTACGTCGtcgatcagaggcgagagtcctcgatgaagaaggatgaaggtttatggtgcggccgtaaatgcaagccaagttctttacggcccttaacacaaaccctaaatgtggattacgctaatattgtcccctaataaagtggcctggaacgatggctggccctggctcgcaggcggagagtattgcgtctgat**tggtttttcccacg**attacaattagccggacagcgggacagacctgacctgacctgaccctcatgctgaccgttccactgccactcggaactgaaggaaacagtggacggacgcaggccagcgctgtggattgtgcactaagttgcttaatccgacgggaaatccaaaacaaatgcgacggcctgcacccgatgatgtggcgtgtcaagttattgaactgactctcgcacaccgatcctatacgatcctatccgatcctgaggcgagcagaggctatcctctacgcgttcctcaggtcgaggcaaggcgcagacctaagccaaagttagaggcacac**cacatgtg**tgtttgcgactggc**gggaaagccc**cattttaaagctgggccaaaacggca**acacatg**gtgcgagcgaccctcatcccggacctggacaggttcacattccattccctggaagaagaaaaaaacaagagaacgtgtacgagaacccggaggaccaaaccaggcaggtcgatggagaggagccacttgtcgggaaaacctccaacaaagaaaaatatcaagtggcattgttcctgatctttcagttaggaattctcagagattcgcaatatcagagattactaatcaaactCCCAGATGGGGTTAGGATATG

**>Dv_brk_NEE_744_bp**

TGTCCGGGCTTATGGATCGtaaagccaagttctacacataaaatgtgctataactggattacgctaatatttgaaccctaataaaaaagaagtgcacgaatgttgcgtctgat**tggtttttcccacg**attacaattagcgggcaggcagccagccaggcaggcagtcgggcgggcaggcgggcgggcgggcggacggacagtcggacggacagacgttcaggtcgcgactaccttgaagccaagtgcggaactgaaggaaacaaaacggcgaacgctgcgacggattgtgcactaagttgcttaatccgacgggaaatccaaaacaaatgccgccggatggtgtacggctgtgtgtgtgtgtgtacgggtgtgtgtgtacgggtgtgtgaggtgttattgaactgacgaagagccctgggctcgagcccagttcgcggcctgagccgctcttttggttgcctggctaagccaaaatgttagcggcacagagggcacaatgcggattcgttgcgggccaggtctgtccagatctacaatatatggcatcggag**cacatgtg**tttggctggac**gggaaagcccc**atactaaagctggccggtggccaa**cacatgt**cttgggcaattgccaggccaataccgtggcacaggttcaatacacagttcaacagaactatgcctgcccaggcaggccctcgtatatcgtataccatttcaagtaaaCCCAAACTTAGCACGGATAATGCA

**>Dm_rho_NEE_871_bp**

CAGTTAAGTGAGTCGCTTTCAGGactcagggcatcatccagatcgcacgatcccatttgcatctgccttctcagaagctgcttgaaagacgcgcccctgtcggatgattagtgctaagatccttgggcaggatggaaaaatgggaaaacatgcggtgggaaaaacacacatcgcgaaacatttggcgcaacttgcggaagacaagtgcggctgcaacaaaaagtcgcgaaacgaaactctgggaagcggaaaaaggacaccttgctgtgcggcgggaagcgcaagtggcgggcggaatttcctgattcgcgatgccatgaggcactcg**ca**t**atg**ttgag**cacatgt**tttgg**gggaaattccc**gggcgacgggccaggaatcaacgtcctgtcctg**cgtgggaaaagccc**acgtcctacccacgcccactcggttacctgaattcgagctcgagtgttttgggtggctgagattgctttggtacggtggctgaccttgccagtgccagtgggtccatgtccgtggagtgctggcaatgcgtgtctggcacaatgaaaacgcagcaatggaggatggaggaactcgacctgagggaa**cgtgggaaa**aaaggatttacgatgaagatgagctgagaaactccgagctgatgacgtgcctggtcatgggcatgtagtgcagcaactgtgttttcgtatctgcttacctgttgcacg**cacatgt**taaaaagtcatcataaatctcagtggacgaggacgaggactctgaatctgtttcgcctctcttttgcgcccagtgaaaatattttcacttttcatgcgggagttttcatgccgccgatgaggatgactGGCCAGGATGGGTATATCTATCTA

**>Dp_rho_NEE_843_bp**

gtTccaGGAAGCTGCTCTACAGACGCGcccctaacgaaacagatcccagatcccgcagatccctggactgggaaaacacacaccacacacacacacacacacagcagtgctcgaggcttggcgtagcttgcggacaagtgcaggacacttttggacagggcagagcagggcagagcagagccgagcagagcggacaaaaagtcacacaaatgaaactctgggaaaagcggaaaagacgcggacgcggctgaggatgaggatgaggcggcggcaggggcaccttgctgtgtgggaaacacaagtggcggaaattcctggccagaaacgaccgtgtccgtcccgacccgacccgtcgcggcc**cacatgt**gtttcg**cacatgt**tgatgg**aggaatttccc**gcgagtgcgcgcggttttaaggcaaaggaatcatcgagtgtcctcccacgcccacagcggcccacagcggcccacgaaggcccacaaaggcaggcagtacctgtgattgaccttgacagggagatggcaatagaaggagggacagtgacgcagggagagccctgccttgggtccactgttttttgtttgatgcgtgtctgccacaatgaacggggcacaagaagaccagaagaacgtgtgaggaaa**cgtgggaaa**aaaggatttacgatggggagggagcgagagagcgggagagaggaacggaatctagagtctgatgtctgatgtctgatgatgtgcctggtcatggtagcagcgtgtatctgtgtatctgtgtcttacctgttgcatgttg**cacatgt**taaaaagtcatcataaaTCTCAGTGCTTCGCTGTGTGTGtgcgcg

**>Dv_rho_NEE_726_bp**

CTGGGAAGTTGCACGAGAGACGCgcctctgcatagcagagcaattgactggatatccttgtgctgggaggacacatgactccggagatgggaaaaacacacgcacgcacacggcgatagaaattaacacgtagtttagcggaactttgtggcaagtgcaacaaaagtcgaagtcgcggacgattcaaatgaaaatctgcaatgctgcggaaggagcaaggacaacccacctgtctatgagtgtgcgagtgtgcgagtgtgtgtgtgtgtgtgcgagtgtgtgtgcgtgcgtgtgtgtgcaacaagtg**cggaaattcct**gaatcg**cacatgt**ggcacg**cacatgt**cgagc**gggaaa**aaaccgctcgatgctcggtccaaggaattccccgagccaagggaagtccgcccaaaacacgcccaaaaggcggcaattatgattacctgtgaccggtgacccggtgaacggtaacccggcgaacagtgactcggtgaacggtgactgtgacttgagtggaggctggctacgtgcctgggacatgcctcccagatttgaacggaa**cgtgggaaa**aaaggatttacgattctgttgccgatgacgtgcccggtcatggtcatggtcatggtcacagtatgtgtgtgtgcgtgtgtgtgtgtgtgtgcgggcttacctgttg**cacatgt**taaaaagtcatcataaatctcaGCGTTGTGCCAGAAGAGTTTCTC

**>Dm_vein_NEE_919_bp**

CAGTTCTGGATCTTCCGAATCACCctgccttcgcgttttgcaccccgtcgctgtggagccatattttctttttagctgacgatttagtccatttcccgctcataatcgcatgaagttgtttgcctccaccgaatggcttaatccgccagatcgatgcgcctgtgttgactcaataattccctaacaactctttttacgcattttattgaaagtgccgaagttagcgggcatttcacttacctg**cgtgggaaaatcga**ctaatctgcgaccgccccgaggagtcagtttttgtttttagagcggtaaaggacaggtaacgggc**cacatgt**ctggc**cggaaattccc**cgttgacccctgaccccgtgtccttatgacgaattcgtcacttggcgtgagcacacctggatttcccaccgcttagccagcggaaattccaaaacacctccggcccactggccctcaaaattgttatatgctctgctacgatgaagcagaagcagaagcagcagtgttttattggcggaagcatccgccaaattgcacccaatctgcagtttgaagtgctcaaaacccccaccgctcccctgtgaatttccgccggccggcaaggtgaccgtgtgctaaaacaaaatttttatatcgaaattgccgccggtcaacgcgcgcgcctgcccaatggccactttaaccacgtttcagcgcgtgttggcctttttggttcccaagctttgtttggcccacacagacagacactctactctgtccgcgtatatgcatgtgtgctcttacactcactcacacacacactcacacacacacaggcacacacaaaagccatttcggacaagttggagttgcatttttagcagcatttttgtttttctgcggcattttattCGTCGCCGCTGGCTACAAATTT

**>Dp_vein_NEE_858_bp**

TGTGGGGCAATATTTTCTTTTTAGCtgacgatttagccaatttcccgatcataatcgccctcgatccggctgccagaggctgcaatgaagaagctgtttggctctttggcatttgtctggtccagtgaatggcttaatctggcgagtgttgattcaataattccccaccaaagtgcttggagaaatctgggaagcaaatctgtcgcctgtcgcctgtcgtcggatacctg**cgtgggaaaatctc**tcgtttcctggactaatctgcgaccgccctaaagagtcaggttccaaaggcccaagcggacaggtaactggc**cacatgt**ttggc**cggaaattccc**atgcgcctcctgtgctgcctctgacctcgtcctgacattgtccttatgacgaattcgtcataattcgttccctactgctctcagggcatttccagcatttgcgcttgccttgcaaaatgatatatgaaaaaaatatgtattattagcggaagaagcgggccaccaaattgcatccaatcatggccaatatctgcccctgccactgccactactactgccagtgccactgcccccagcttcggccactgtgccccatcctcttctttctctcaggctgctgccggccggcaaggtgaccgtgtgctataacaaaatttttatatcgaaattgccgctggtcaacgcgcgcgcctgcccaatggccacgttaagcgcgtgttggcctttttggttcccaagctttgtttgccacaagcacagataaagatacacacacacacacacacacacgcacgcacacacacagagtcacagatacagatacggccccgctCAGACAAGTTGGAGTTGCATTTTTAG

**>Dv_vein_NEE_836_bp**

**CA**t**ATGT**TGCCCCTTTGCTGTTGCtgctgcttgtggcaggcaatattttctttttagctgacgatttagccgatttcacgatcataatcgcattgctgctgcagctgatgatgttgttgtcacttaaatcgcttaatctgcgcaattcaattgagttcttaacttgttaatggaattgatagaagctcagtgcctggccggctgtttcggtcagtcaagggctgcacacacaccctacctg**cgtgggaaaatgtc**cattgcgctctcgggctaagctgttttggttggcgggcacaggtaacaggcggtagggcaacgttgcaaccggcaaatgtggctgtgc**cacatgt**ttgtgc**cggaaattccc**cgtgcacgccttaccgcctgcctttttatgttgaaatttttcctatcacttggcgggtttttccgggccactggcggaaattccacaatgtcgccactggccacaggcagaaagtgcaaccaaacgcgcttacaaaaattattataaaatgtgtattattaaatgccaaattgcgtccaatcatccgcagttctctgccggccggcaagctgaccgtgtgctaaacaaaaatcaaaaaaaaaaaaaaacagccaaaattgccgctggctgctgcacaaacatgcgagcgcgcgcgcgggctccaactggccactggggcaagctgagcgcgtgtttgtcttttcggttcccatgcacacacacacacacacacacacacacacagacacactcacgcacacacagtttcaagttgaagttgcatttttagcagcatttttgtgcgGCATTTTATTCGTCGCCGCTGACA

**>Dm_vnd_NEE_1020_bp**

GGAAGATTGGGCGTTGAAAGCagaatctgcagaatacgcaagaccattaattaattctcctaatctaataatttggaagattgggcgttgaaagcagaatctgcagaatacgcaagaccagccatactttgcaattgccttcgcctaagctccgacttaaaatcacaccctgcgagcctctgcctccatattagtgtttagatcccatatagctgatggacttgtccggctaatcctggtagctctttaaattaaccaagcgggcacagcgcgcaagtacaggacacagggtataattccccgcctctttgatctggcaagtactcaaggtcctggcgaatggcggttgggaaattctggcttgttgttcgaccctggcttagaaattcccgtaggtgagagccagggaaaccccaatcgggaatg**acatgtg**tacgacagacatgggactcagatgccttcgagatactggcgtcacactgtctggcaatgggatttccgctcaggaggacggggaatgcccgtgtagcctgtccatag**cgtgggaaattcgc**gagtcggggtcttcgggaaaactcgaaat**gggaaaaccgg**aagcaagcaaacttgcgcca**acatgtg**gcacgacctgtttcgacccgtaaagagtccctgctgacctgtgctgacctgcactgacccgaccaggtagctgcgatccttacgaggcggatttgcgtttaattgttgatggtattaggcaaatcaaaactcggggtctgaccgggactaggtgtcaataatccagcgatttgggtgcacttattcaaagttaattccgggggaaatgtgcgcgttttcggttccgaagcatgcctgcaggatgcacaccccccacctccttatcttcttaacaacggcaagtgcaaaaatctgtgaaagtcagagcgctacaggtagtgcaggtagtttcctttgcatatcccgaccaacagggacctccttttgttaaaccttcCGGCCATTCACACGATTGACACA

**>Dp_vnd_NEE_1305_bp**

GTATTTCGAAGATGCATTTGTTTGCttatgtaatttttaaacgccagtttttctcttgcaatgctccgaatctcccacaaaagaagttctgccggtatctagatatttgagctttagattccgcagacttacacttttctaaccgctgaagtaaacaaccaaatagggttccattactatctaaagagatccccggcttgatcggtccttactctacgcataagccgcggtttgccttcgcctaagcttcggctcatagtcacacccatgagcgagcccctgccctagtcctggccatgcccagtactatgtacatggctagctaatggactttgctcactaatccaatgaggccacttgcagagccaagttagccaaatcctgccgagaagcatcacggcatagcacatcacaagaaaacggcgaatatgggacgtatttcccccgtttctggtcgagcaagtactcaaggtccttgcttttggatcgaagaaattcagcttgatttaccctgtgctggcctggcttagaaattcccatccatcgatggacagtaggtgagagtggtca**gggaaactcca**atgacgggcca**acatgtg**tgcggcagacagcagaggactcggtgcagattcgagacaccgggctgacactgccgctgacgacagggaagatgcagacgacgacgtcacactgtctggcgaagggaattccctcggagaaaa**acatgtg**tctccgtctttgtcccactatcgtaccaacgctgg**cgtgggaattt**tggaaaattcaatgtggaaaaaccggaagcagcgcgcgaagaaacttgcgcca**acatgtg**gcacgacctgagccgacctgaaccgacctgacccgatccgatctgatcttcggatggacaggtagatggcaaccaacaggtcccagcggtggtcagtggttggtctaaagcagatttgtgtttaattgttgatggtattagtggaaaatcaaattttcattagcctctgggcccagtacaggatttggcaagtggaagcccaaaaggaaaaggaaaggaaaggccactgagactaggtgtcaataatcctgcgatttgggtgcacttaatcaaagttaattctcgcccgagaatttttcagggagatcagatcagatagatcccaatgtctcagatagttcgctcgctccctctgcaaaaatcgcaatctgcgaaagtcagggcatcggcaggtagcacaggtagaccggtagaacaggctgtattcgagccacagtgttaaagcCTTTGTTGGACCTTCCGGCCATTCA

**>Dv_vnd_NEE_1093_bp**

GGCCCGTGTCTGTGTTATTTTAGGcctcggtcagctaatgaactttgccttctaagccagccagccagcaactgtaacgagctatgccactgagccaaggcaatccaatcaaggcacttactccctaaacattgtccatctacttgtttcgggcacttgccccgcgcagagcctgctaaagcaaattacccaaatcctgaagcagccttaagatgtgacagtgaccacacgtaattcccccagacaagatccgcagatttgctgggctagtgcatgctcaaatatgctcaaggtcctgtaattgtgtgcagcactcttgtccgacattgattggcgatcgcagctcaagtggcaaaagacgcaacggacgtcgatctgtttggcctggctggcttagaaattcccataggtgagttgca**gggaaacccc**aacctggca**acatgtg**tgcagcagacgacagcatcagcaacagcaacagtaacagcagcggcgacgtggacggcgtcacactgtctggtcgaaggccgggacgagtgcaaagggaattccgctcagacatgcggggaatgccccgcgtcctgatggacctgagcg**cgtgggaatttcgc**gcgcacaattt**gggaaa**actcagcgtggaaaaaccggaagcgagagcgctttttacttgcgcca**acatgtg**gccgaaaaatggttagacctgaccaaagagacccgcgcccgatcgcccgatagtccaggtagtgcagcggtgcaccaaaagcggatttgagtttaattgttgatggtattagtggaaaatcaaatttgcattagccctagcttagcccaaaactaaaactgaaactgaactgaaactgggtctgaaactgtgactgggcttttggttttgcaactgggactaggtgtcaataatccagcgatttggctggcacttaatcaaagttaattccgagcaagtgtgaaaaatctaagcccgagtcgcagctcggtgcaattttcgtcaaagtcagcgcgtcaggtacttgctgcttggccaggtagaaatttagacccgaaaatgcagtgtacgtCCATTGTGTTGCTTTCCGGCCAT

**>Dm_sog_NEE_550_bp**

TGTTTATGGCAGCCAATTGATGCCGActgacctgtgtgtgtgtgtgtgtgtgtgtggaagctcaggatggacagattcccgggtttcagcggaacaggtaggctggtcgat**cggaaattccc**accata**cacatgtg**gctataatgccaacggcatcgaggtgcgaaaacagatgcagcctcataaaaggggcgcagataaggtcgcggttg**cgtgggaaaagccc**atccgaccaggaccaggacgaagcagtgcggttggcgcatcattgccgccatatctgctattcctacctgcgtggccatggcgatatccttgtgcaaggataaggagcggggatcataaaacgctgtcgcttttgtttatgctgcttatttaaattggcttcttggcgggcgttgcaacctggtgctagtcccaatcccaatcccaattccaatccgtatacccgtatatccaatgcattctacctgtcct**gggaatttccg**atttggccgcacc**ca**t**atg**gccacggatgcgtgagagtgCTCTCCGTGCGATTCTAGATCATC

**>Dv_sog_NEE_871_bp**

CACGGAAACAACGAGGCAA**CGgaaacacgt**aaattcccataatgtgagcggatcataaacaccaacacaacacaagacaacacagccaagcagaaagtgcgaaaaacagatgcgttgtgcggtgcggtgcggtacggtgcggtgcgatggctcataaaaggggcacaagagataagcgagattgggc**cgtgggaaaagccc**aaaaaccgtaccgacgacagcaacctgtagggaacggatcctgttcgctggatggataatggcggcgtcataaaacgcagtcgtcctttgtttatgctgcttatttaaattggcttcttggcgggcgttgcgcggccaaaaccaaaaccgagacgcagattgagagagagagaaagagagagagagggagacggcaacctaaccgatcttaactttaacctgacctgagatctgagggtctgcctgtgggctacctgtgcc**ggggaattccc**gtaacctttaacc**ca**t**atg**gctgggccgctgccgtgagagtacgggcaacaggggcagacaatctgtcgcatttctggatcatcatggcccagccaaatagcgcgttattgttagcaattggttttcaagaaaacacacacacacacacacacaacggggttttcccacaatgcactcggtgtgtgtgtgtgtgtgtatgtgtgtgtgtgcacgggcccagccaaaacgtgcgtcatctgttgctcctggacgcgacgcgcacttggaacgaacgaacctgggcaggattacagattgcgacaatgcgttcactggaaacagccgtctgcgtgagctgttttgtgggttttggttttggtttcggtttcggatttgGATTCGGTTTCCGTTTCGCcaT
